# Any closer to successful therapy of multiple myeloma? CAR-T cell is a good reason for optimism

**DOI:** 10.1186/s13287-021-02283-z

**Published:** 2021-03-29

**Authors:** Faroogh Marofi, Safa Tahmasebi, Heshu Sulaiman Rahman, Denis Kaigorodov, Alexander Markov, Alexei Valerievich Yumashev, Navid Shomali, Max Stanley Chartrand, Yashwant Pathak, Rebar N. Mohammed, Mostafa Jarahian, Roza Motavalli, Farhad Motavalli Khiavi

**Affiliations:** 1 Department of Hematology, Faculty of Medicine, Tabriz University of Medical Sciences, Tabriz, Iran; 2 Department of Immunology, Faculty of Medicine, Zanjan University of Medical Sciences, Zanjan, Iran; 3 Department of Physiology, College of Medicine, University of Suleimanyah, Sulaymaniyah, Iraq; 4 Director of Research Institute “MitoKey”, Moscow State Medical University, Moscow, Russian Federation; 5 Tyumen State Medical University, Tyumen, Russian Federation; 6 Department of Prosthetic Dentistry, Sechenov First Moscow State Medical University, Trubetskaya St., 8-2, Moscow, Russian Federation 119991; 7 Department of Immunology, Faculty of Medicine, Tabriz University of Medical Sciences, Tabriz, Iran; 8 Immunology Research Center, Tabriz University of Medical Sciences, Tabriz, Iran; 9 DigiCare Behavioral Research, Casa Grande, AZ USA; 10 Faculty Affairs, Taneja College of Pharmacy, University of South Florida, Tampa, FL USA; 11 Faculty of Pharmacy, Airlangga University, Surabaya, Indonesia; 12 Bone Marrow Transplant Center, Hiwa Cancer Hospital, Suleimanyah, Iraq; 13 Toxicology and Chemotherapy Unit (G401), German Cancer Research Center, 69120 Heidelberg, Germany; 14 Stem Cell Research Center, Tabriz University of Medical Sciences, Tabriz, Iran; 15 Department of Virology, Pasteur Institute of Iran, Tehran, Iran

**Keywords:** Multiple myeloma, Adoptive cell therapy, CAR-T cells, Hematological malignancy

## Abstract

Despite many recent advances on cancer novel therapies, researchers have yet a long way to cure cancer. They have to deal with tough challenges before they can reach success. Nonetheless, it seems that recently developed immunotherapy-based therapy approaches such as adoptive cell transfer (ACT) have emerged as a promising therapeutic strategy against various kinds of tumors even the cancers in the blood (liquid cancers). The hematological (liquid) cancers are hard to be targeted by usual cancer therapies, for they do not form localized solid tumors. Until recently, two types of ACTs have been developed and introduced; tumor-infiltrating lymphocytes (TILs) and chimeric antigen receptor (CAR)-T cells which the latter is the subject of our discussion. It is interesting about engineered CAR-T cells that they are genetically endowed with unique cancer-specific characteristics, so they can use the potency of the host immune system to fight against either solid or liquid cancers. Multiple myeloma (MM) or simply referred to as myeloma is a type of hematological malignancy that affects the plasma cells. The cancerous plasma cells produce immunoglobulins (antibodies) uncontrollably which consequently damage the tissues and organs and break the immune system function. Although the last few years have seen significant progressions in the treatment of MM, still a complete remission remains unconvincing. MM is a medically challenging and stubborn disease with a disappointingly low rate of survival rate. When comparing the three most occurring blood cancers (i.e., lymphoma, leukemia, and myeloma), myeloma has the lowest 5-year survival rate (around 40%). A low survival rate indicates a high mortality rate with difficulty in treatment. Therefore, novel CAR-T cell-based therapies or combination therapies along with CAT-T cells may bring new hope for multiple myeloma patients. CAR-T cell therapy has a high potential to improve the remission success rate in patients with MM. To date, many preclinical and clinical trial studies have been conducted to investigate the ability and capacity of CAR T cells in targeting the antigens on myeloma cells. Despite the problems and obstacles, CAR-T cell experiments in MM patients revealed a robust therapeutic potential. However, several factors might be considered during CAR-T cell therapy for better response and reduced side effects. Also, incorporating the CAT-T cell method into a combinational treatment schedule may be a promising approach. In this paper, with a greater emphasis on CAR-T cell application in the treatment of MM, we will discuss and introduce CAR-T cell’s history and functions, their limitations, and the solutions to defeat the limitations and different types of modifications on CAR-T cells.

## Introduction

Multiple myeloma (MM) is the second most prevalent hematological cancer that attributes to a plasma cell malignancy specified by the increased proliferation of mutated plasma cells. Microenvironment variation in bone marrow, plasma cell mutations, and immune surveillance disability are the main causes for monoclonal gammopathy of undetermined significance (MGUS) and multiple myeloma [1, 2]. In this regard, several therapeutic approaches have been assigned for treating MM patients, including immunomodulatory drugs (IMiDs), monoclonal antibodies, donor lymphocyte infusions (DLIs), and allogeneic stem cell transplantation (allo-SCT). However, the curability and prognosis of the patients mostly remain poor in relapsed and refractory (RR) MM patients [1, 3]. Approximately, 3 years of median overall survival have been reported in higher stage and high-risk cytogenetics patients [4]. Currently, significant steps have been taken towards the development of immunotherapy-based drugs for treating patients with multiple myeloma. Monoclonal antibodies, checkpoint inhibitors, antibody-drug conjugations, bispecific T cell engagers (BiTEs), and adoptive T cell therapy (ACT) are examples of immune-based therapies that have been expanded for MM treatment. Beneficially, daratumumab and elotuzumab are FDA-approved monoclonal antibodies that have been indicated to strengthen the immune system to target the MM cells [5, 6].

Nowadays, the ACT has been shown impressive results in cancer treatment among immunotherapy approaches by boosting the immune system response. The ACT is performed to transfer the manipulated autologous cells into patients’ bodies [7]. During the last decade, genetically engineered chimeric antigen receptor (CAR)-T cell therapy has been identified as an advanced subgroup of ACT for treating cancers, infections, and allergic disorders [8, 9]. Hopefully, CAR-T cell therapy has illustrated the beneficial implications in hematological malignancies [10]; however, barriers in solid tumors cause CAR-T cells to become ineffective [11]. CAR-T cells are generated by transferring the manipulated gene into T cells. CAR-T cell comprises the recombinant antigen receptor that binds to the target antigens, and the T cell signaling portion which activates the T cells. Besides, this synthetic CAR-T cell possesses the antigen recognition ability in a non-MHC-dependent method. In comparison with conventional T cells expressing TCR, CAR-T cells are characterized by several benefits, including simple structure, targeting various types of antigens to overcome the tumor escape, more significant anti-tumor cytotoxicity, proliferation as well as persistence in determining tumor sites [12, 13].

Since the hypothesis of CAR-T was firstly presented in the late 1980s, many supported preclinical and clinical studies have been carried out to evaluate the capability of engineered T cells in diverse hematological malignancies. CD19, CD20, CD30, and CD138 are the most predominant target antigens found in different species of hematological cancers. CD19, a prevalent surface marker on B cell, is used to engineer the anti-CD19 CAR-T cell which was approved by US Food and Drug Administration (FDA) for treating diffuse large B cell lymphoma (DLBCL) and acute lymphocytic leukemia (ALL) diseases [14, 15]. CAR-T cell therapy as a promising therapeutic method for multiple myeloma was first developed for ALL, CLL, and DLBCL malignancies.

The success of immunotherapy approaches, like CAR-T cell therapy, requires the severe and selective expression of target antigens on tumor cells along with their non-expression on normal cells. The clinical studies have identified several numbers of target antigens expressed on abnormal plasma cells in multiple myeloma, including CD19, CD38, CD138, SLAMF7, kappa light chain, B cell maturation antigen (BCMA), and SLAMF7. In the current study, we aimed to demonstrate a comprehensive overview of the immunotherapy approaches for treating multiple myeloma by focusing on up-to-date knowledge on various CAR-T cells designed for targeting different target antigens on abnormal plasma cells in multiple myeloma.

## Immunotherapy therapeutic approaches in multiple myeloma disease

### Allogeneic stem cell transplantation (allo-SCT)

The allo-SCT is the first line cell therapy method in multiple myeloma, leading to longstanding progression-free survival (PFS), and curative graft-versus-myeloma (GvM) influences. Despite the potent remedial role with up to 50% remission rate, the allo-SCT is limited due to the upward trend of non-relapse mortality (NRM) rate of approximately 40–60% [16]. There are also significant adverse effects related to allo-SCT, including greater morbidity and mortality rate, infection risk, immunosuppression, and graft-versus-host disease (GVHD), all of which have restricted its application [17].

### Monoclonal antibodies

Monoclonal antibodies (mAbs), as pivotal therapeutic approaches, have been designed to overcome exclusively target antigens overexpressed on abnormal plasma cells in multiple myeloma, but not on normal cells [18]. To eradicate the tumor cells, antibodies trigger the antibody-dependent cellular cytotoxicity (ADC) along with the complement-dependent cytotoxicity (CDC), both of which lead to the secretion of cytotoxic components after binding to targets [19]. In this regard, there are no pieces of evidence showing their involvement in tumor cell eradication in MM unless when used in combination with IMiDs, proteasome inhibitors, and corticosteroids [20]. mAbs in multiple myeloma have been generated against CD38, CD40, CD56, CD74, CD138, SLAMF7, KIR, and PD-1 target antigens, two of which are applicable and FDA-approved for treating patients with MM, including daratumumab (Anti-CD38 mAb) and elotuzumab (Anti-SLAMF7 MAb) [21].

### Antibody-drug conjugates (ADCs)

Antibody-drug conjugates (ADCs) are composed of mAbs conjugating to anti-tubulin cytotoxic factors. These antibodies detect and subsequently bind to the target antigens which accelerate the eradication of tumor cells through the toxin agents releasing into the tumor microenvironment. In phase I/II studies, an anti-CD138 ADC (indatuximab ravtansine) along with lenalidomide and dexamethasone have been shown promising results with approximately 78% objective response rate (ORR) [22].

### Bispecific T cell engagers (BiTE)

Bispecific T cell engagers (BiTE), or bispecific antibodies, are constituted of a recombinant antibody with specificity for two distinct epitopes, such as CD3 on the T cells and target antigen on the myeloma cells. T cell activation and functionalization are mediated by simultaneous transplantation between two epitopes and BiTE on tumor cells [23]. It has been revealed that the constant development of this approach provides the redirection of a large number of T cells into the tumor microenvironment. CD138- and CD3-specific antibodies are the example of BiTE produced for MM treatment [24].

### Checkpoint inhibitors

Programmed cell death protein ligand-1 (PD-L1) is expressed on tumor cells, applying the inhibitory function against the effector role of the T cells by binding to PD-1 which is expressed on T cells [25]. PD-L1 is upregulated at both relapse or refractory phase of MM. A great population of PD-L1^+^ MM cells along with their high resistance to anti-myeloma factors has been demonstrated in patients with MM [26]. Nivolumab, an anti-PD-L1 monoclonal antibody, has shown objective responses in phase I clinical trial with approximately 63% responsiveness among 27 patients, and overall survival of 11.4 weeks in relapsed/refractory MMs [27]. Moreover, in phase II of a clinical trial, beneficial outcomes of integrative therapy have been implied in patients with MM using anti-PD-L1 antibodies (pembrolizumab and pomalidomide), and dexamethasone [28]. Since there are no reported controversial reactions of the checkpoint blockade immunotherapy in treating patients with multiple myeloma, it may be successful in combination with other therapies.

### Vaccines

Several known peptides or dendritic cell-based vaccines are used to build long-term memory against disease recurrence by boosting host immunity and increasing tumor-specific lymphocytes [21]. In this regard, peptide-based vaccines have been administered for MM patients to induce the myeloma-associated antigens on plasma cells, resulting in the expansion of effector T cell repercussions. Multiepitope peptide vaccines have been developed to overcome remarkably various target antigens. For instance, CD138, X-box binding protein 1 (XBP1), and connecting segment 1 (CS-1) peptides are multiepitope vaccines that have elicited an influential effect on the T cell activity, resulting in the eradication of myeloma cells in vitro [29]. This kind of vaccine revealed robust immune responses in an examination of smoldering MM patients [30]. Furthermore, a dendritic cell-based vaccine has been shown to be useful in treating patients with MM. This method can be established by the fusion of patient-derived myeloma cells with autologous dendritic cells. Promising results have implied that usage of the dendritic/myeloma cell-based vaccine in the phase I/II trials conducts to an increase in the frequency of CD4b^+^ and CD8b^+^ myeloma specific T cells [31, 32].

### Adoptive cell therapy (ACT)

The ACT has emerged to promote the performance of effector cells in targeting antigens and eradicating the tumor cells by ex vivo manipulations. Also, the ACT isolates autologous T cells by leukapheresis, reinfusing the ex vivo engineered and cultivated cells into patient’s bodies. Three main ACT-based approaches have been comprised of several forms, including T cell receptor (TCR)-engineered T cells, autologous tumor-infiltrating lymphocytes (TILs), marrow infiltrating lymphocytes (MILs), γδT cells, CAR (chimeric antigen receptor)-T cells, and NK cells. They have been specifically developed for MM immunotherapy [33]. In this study, we predominantly focused on different CAR-T cell therapies in MM malignancy.

### Chimeric antigen receptor-T cell

Broad utilization of the engineered chimeric antigen receptor (CAR)-T cells have taken a great step forward in cancer immunotherapy, especially in hematological malignancies. So far, CAR-T cell therapy has been intemperately studied in varying preclinical and clinical investigations [34–38]. To engineer CAR-T cells, T cells should manipulate for expressing CAR genes by viral vectors [39]. There are also several examined transferring techniques, including CRISPR/Cas9 gene-editing strategy [40], electroporation [41], and liposomes [42]. CAR-T cells could significantly dominate conventional TCR restrictions, for instance, HLA-dependent limitations, defined specificity for peptide antigens, limited applications, lower cytotoxicity, persistence, and proliferation.

Modified TCR-like molecules (CAR) have been comprised of single-chain variable fragments (ScFv) as an extracellular domain, spacer (CD4, CD8, or IgG4 Fc), a transmembrane portion, and signaling domain (CD3ζ) with or without the presence of costimulatory domains (CD28, 4-1BB, or OX40) [11, 43]. The ScFv originated in light and heavy variable chains of a monoclonal antibody that diagnoses target antigens on tumor cells in an MHC-independent way. Antigen recognition elicits CAR-T cell cytotoxic function through dominating the signaling domains [13, 44, 45].

**Fig. 1:**
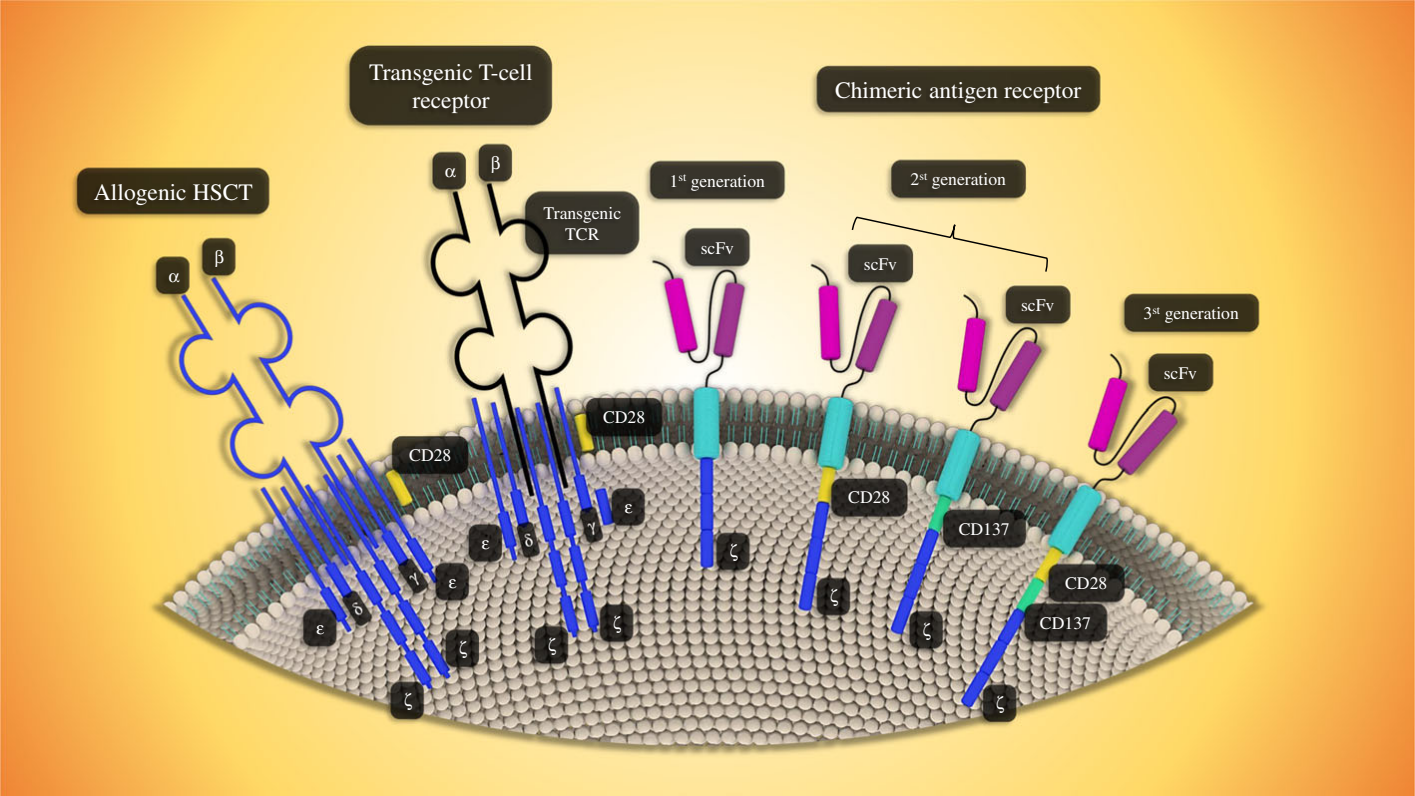
Allogenic, transgenic, and chimeric antigen receptors. In this figure, every kind of receptor has been shown, however, chimeric antigen receptor has been made of three generations which are shown

**Fig. 2:**
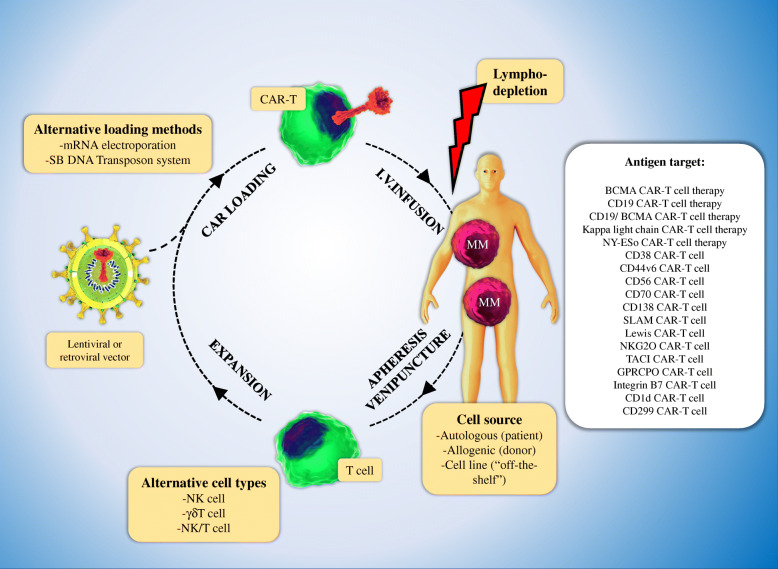
Isolation, engineering, expansion, and administration of CAR-T cells in MM. CAR-T cells from patients with MM are usually produced from autologous T cells collected through leukapheresis (Stage 1). Allogeneic donor or cell lines can be used apart from the autologous T cells (11). γδ T cells, NKT, and NK are applicable as alternative lymphocyte subsets to generate CAR-T cells. The next step performs in ex vivo which the cells are directed to be expanded (stage 2) and are loaded with a vector-encoding CAR gene (stage 3). Non-viral methods such as electroporation or sleeping-beauty can be done. IV injection of CAR-loaded T cells into the patients who usually receive chemotherapy before the lymphatic injection is the next step (step 4). Various MM antigens as CAR-T cells’ targets have been shown

**Fig. 3:**
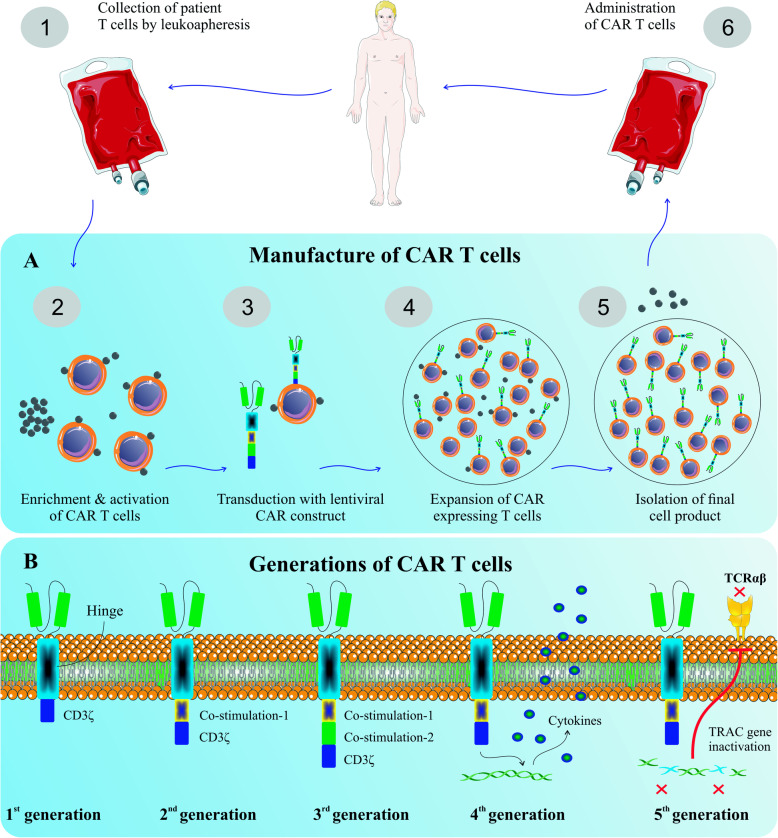
The principle of CAR-T, its preparation, and four engineered generations of CAR-T cells

CAR-T cell is clustered into four generations, composed of ScFv and CD3ζ signaling domains. Additionally, there are the second and third generations that possess the one and two costimulatory molecules, respectively. T cell Redirected for Universal Cytokine Killing (TRUCK) is a fourth-generation CAR that is identified by secreting cytokines or biological factors in which their genes are inserted into TRUCK construction by transduction. Interestingly, it has been revealed that efficacy, cytotoxicity, proliferative capability, and persistence of the third generation of CAR-T cells have significantly been improved in comparison with the first generation (Fig. 1) [12, 46, 47]. The principle of CAR-T, its preparation, and four engineered generations of CAR-T cells have been shown in Figs. 2 and 3.

## CAR-T cell therapy in multiple myeloma

Strikingly, the effectiveness of CAR-T cell therapy is well embraced for treating various hematological malignancies, mostly in acute and chronic leukemia, lymphoma, and multiple myeloma [40, 48]. Indisputably, CAR-T cell therapy would be considered one of the promising and powerful tools despite some important concerns about its efficacy and safety issues. CAR-T cells directed against CD19 are common types of CAR-T cells that have emerged for diverse hematological cancers, mostly leukemias and lymphomas [36, 37, 49, 50]. Furthermore, CAR-T cells targeting CD22 and CD20 have been investigated in ALL [51, 52] and relapsed/refractory NHL, respectively [53, 54]. Anti-CD22- and anti-CD20-CAR-T cell therapies have demonstrated a considerable anti-tumor efficacy. Also, CS-1, CD30, CD38, and CD138 are the other target antigens that can be targeted by CAR-T cells in different malignancies.

**Fig. 4:**
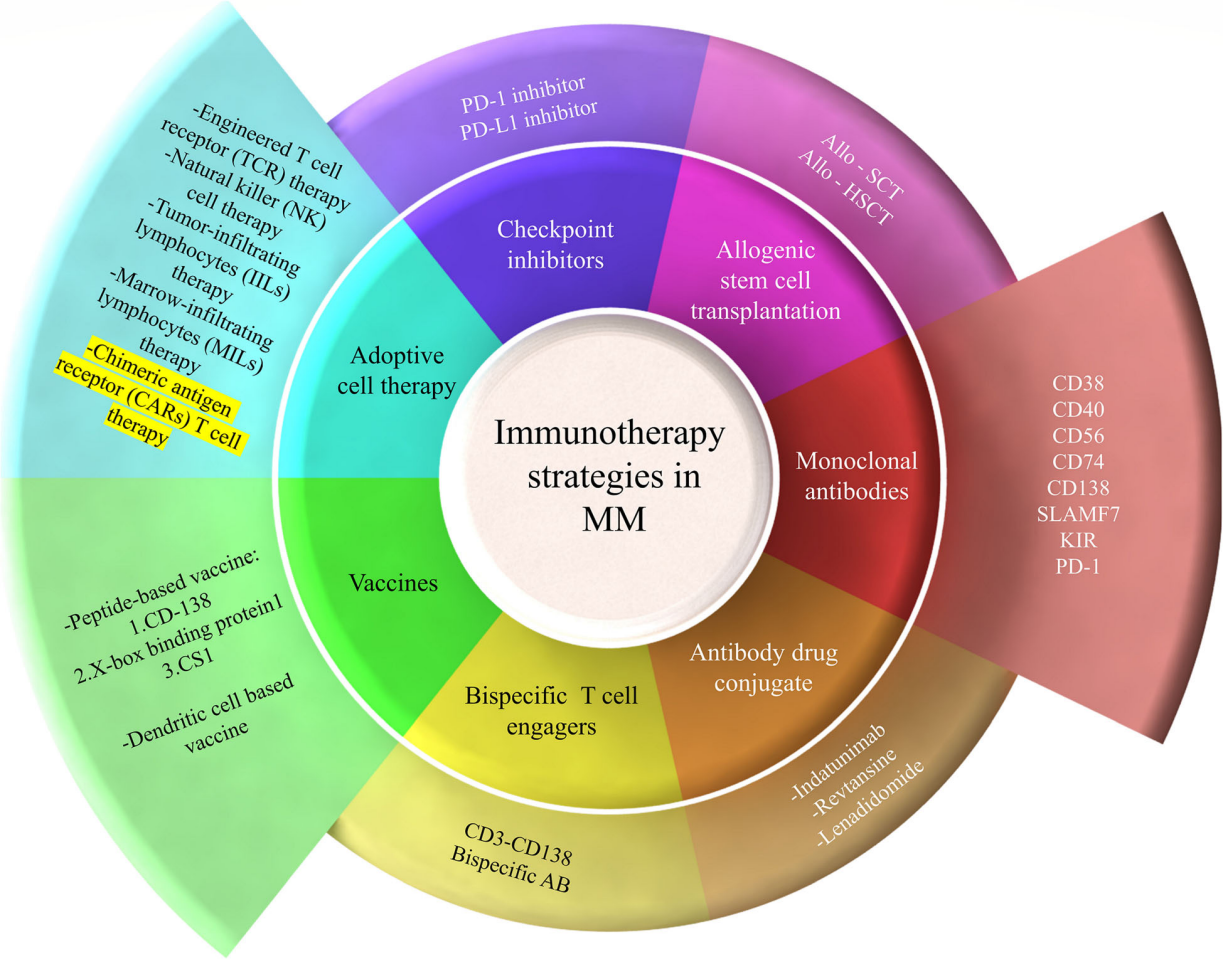
Different immunotherapeutic approaches in MM

CAR-T cell therapy has also been improved to treat patients with multiple myeloma or other hematological malignancies. Previous findings implied that potential and appropriate antigens expressed on target cells can be contributed to procreate the specific CAR-T cells with high efficacy. Antigens that are associated with MM and can be targeted by CAR-T cells are included CD19, CD38, CD138, BCMA, Kappa (κ) light chain, SLAM7, NKG2D, and GPRC5D. Here, we elaborate on overviewing the designed CAR-T cells against the mentioned antigens, and the efficacy of CAR-T Cell therapy in multiple myeloma (Fig. 4).

### BCMA CAR-T cell

B cell maturation antigen (BCMA) refers to a member of the tumor necrosis factor (TNF) family, specifically presented on B cells, normal and malignant plasma cells [55]. Principally, there is a close relationship between BCMA expression and several pivotal factors, comprising of transmembrane activator cyclophilin ligand interactor (TACI), calcium modulator, and B cell-activating factor of the TNF family (BAFF) receptor. BSMA argues the survival of B Cells at different development stages by exerting the BAFF, and proliferation-inducing ligand (APRIL) factors [56]. BCMA plays a substantial role in developing the multiple myeloma cells and considers a proper target antigen by anti-BCMA antibody-drug conjugate (GSK2857916), bispecific T cell engager (BiTE) (AMG420), and CAR-T cell therapy [57, 58]. Pieces of evidence have shown that BCMA has remarkable potential to be targeted by CAR-T cell with less on-target/off-tumor toxicity, and antigen-dependent diminution in the expansion of CAR-T cells [59]. As a disadvantage, recognition of BCMA on MM cells by BCMA CAR-T cells would prevent through blocking role of soluble BCMA secreting by tumors into the blood circulation and peripheral tissues [60].

Up to now, several ongoing or completed studies have been implemented to investigate the various types of BCMA CAR-T cells against MM. The first clinical trial of BCMA-targeted CAR-T cell (NCT02215967) has been conducted by The National Cancer Institute, enrolling 24 patients with MM. The murine single-chain variable fragment (ScFv), CD3ζ (signaling domain), and CD28 (costimulatory domain) were the components of an anti-BCMA CAR-T cell. As a result, minor cytotoxicity was observed using the minimum dose of CAR-T cell (0.3–3.0 × 10^6^ cells/kg) along with a 20% overall response rate (ORR). In return, significant anti-tumor function with 81% ORR and severe cytokine release syndrome (CRS) were reported infusing the highest dose of CAR-T cell (9 × 10^6^ cells/kg) [61] (Table 1).

**Table 1:** BCMA-targeted CAR T cell clinical trials in multiple myeloma

Signal domain	ORR	Efficacy				Side effect		Conditioning	Registration code
		CR	sCR	PR	VGPR	CRS	CRES		
CD28	20%	–	–	1	1	Gr3/4, 38% (high dose)	Gr3/4, 19% (high dose)	CP/Flu	NCT02215967
CD28	87%	–	–	–	–	Gr3, 14%	–	CP/Flu	ChiCTR-OPC-16009113
4-1BB	85%	3	12	4	9	Gr1/2, 70%	Gr1/2, 39% Gr4, 3%	CP/Flu	NCT02658929
4-1BB	86%	–	1	2	3	Gr1/2, 50% Gr3, 12.5%	Gr4, 12.5%	CP	NCT03274219
4-1BB	88%	39	–	8	3	Gr1/2, 83% Gr3/4, 7%	Gr1, 1.8%	CP/Flu	NCT03090659
4-1BB	88.2%	–	13	–	2	Mild, 10 Severe, 6 Very severe, 1	–	CP/Flu	ChiCTR-ONH-17012285
4-1BB	89%	1	1	5	1	Gr2, 1	–	CP/Flu	NCT03288493
4-1BB	100%	1	2	1	2	Gr1/2, 75%	Gr1/2, 38%	CP/Flu	NCT03430011
4-1BB	100%	3	–	4	6	Gr1–3, 1	none	CP/Flu	NCT03915184
4-1BB	64%	–	–	–	–	Gr1/2, 40% Gr3, 20%	Gr2, 10%	CP/Flu	NCT03070327
4-1BB	100%	4	–	4	1	Mild DLT	–	CP/Flu	ChiCTR1800018137
4-1BB	79%	3	4	–	2	Mild	–	CP/Flu	NCT03093168
4-1BB	48%	1	1	5	5	Gr3/4, 32%	Gr3/4, 12%	CP or none	NCT02546167
4-1BB	100%	1	–	3	–	Under Gr3	–	CP/Flu	NCT03661554
4-1BB	95%	3	9	3	5	Gr1–2, 86% Gr3, 5%	–	CP/Flu	ChiCTR-OIC-17011272
OX40, CD28	80%	–	1	2	1	Mild	–	Bu-CP + ASCT	NCT03196414
OX40, CD28	100%	3	–	–	6	Mild	–	CP/Flu	NCT03455972

*BCMA* B cell maturation antigen, *CAR* chimeric antigen receptor, *ORR* overall response rate, *CR* complete response, *sCR* stringent complete response, *PR* partial response, *VGPR* very good partial response, *CRS* cytokine release syndrome, *CRES* cell related encephalopathy syndrome, *Gr* grade, *DLT* dose-limiting toxicity, *CP* cyclophosphamide, *Flu* fludarabine, *Bu* busulphan, *ASCT* autologous stem cell transplantation

#### bb2121 anti-BCMA CAR-T cell

Another clinical study has examined the anti-BCMA CAR-T cell (bb2121) has been composed of anti-BCMA ScFv, CD3, and 4-1BB domains in 33 RRMM patients (NCT02658929). Eighty-five percent ORR was reported among 12 patients, while side effects showed 76% CRS and 42% neurotoxicity [62, 63].

#### bb21217 anti-BCMA CAR-T cell

The bb21217 anti-BCMA CAR-T cell is another next-generation CAR that is evaluated in MM patients. A phase I dose-escalation clinical study on bb21217 (NCT03274219) showed that 7 RRMM patients who were treated with 150 × 10^6^ CAR-T cells had a significant expansion of CAR-T cells, five of which diagnosed with grade 1–3 [64].

#### LCAR-B38M anti-BCMA CAR-T cell

Bispecific CAR-T cell (LCAR-B38M) has been devised to target VHH1 and VHH2 epitopes of BCMA and investigated in advanced RRMMs by a multicenter study (NCT03090659). Although findings have revealed 88% ORR and 68% complete responses (CR) in patients who were treated with LCAR-B38M anti-BCMA CAR-T cells, some adverse effects, including CRS, leukopenia, thrombocytopenia, and pyrexia, have been reported [65].

#### P-BCMA-101 anti-BCMA CAR-T cell

P-BCMA-101 is identified as a novel product of CAR-T cell containing fully humanized anti-BCMA ScFv extracellular domain, CD3ζ, and 4-1BB signaling domains. The structure of CAR-T cell shows more potent and has a high level of expression along with less immunogenicity which is provided by applying a transposon system instead of viral vectors. In a phase I clinical trial study (NCT03288493), the efficacy and safety of P-BCMA-101 CAR-T cells were assessed in patients with RRMM in which approximately one of twelve patients indicated CR, and one of them was diagnosed with grade 2 CRS [66, 67].

#### MCARH171 anti-BCMA CAR-T cell

Another next-generation anti-BCMA CAR-T cell called MCARH171 has been investigated in patients with RRMM in a phase I dose-escalation trial (NCT03070327) study. This type of CAR-T cell has consisted of a humanized anti-BCMA ScFv, CD3ζ, 4-1BB, and a truncated epidermal growth factor. Four doses of CAR-T cells (72 × 10^6^, 137 × 106, 475 × 10^6^, and 818 × 10^6^) were administrated to patients, resulting in approximately 64% ORR. As an adverse impact, up to 40% of patients were diagnosed with 1–3 grade CRS [68].

#### BRD015 anti-BCMA CAR-T cell

A phase I clinical trial study (ChiCTR-OPC-16009113) examined the proficiency of BRD015 anti-BCMA CAR-T cells in 28 RRMM cases. BRD015 has been composed of murine anti-BCMA ScFv, CD3ζ, and CD28 domains. It was administrated with 5.4–25.0 × 10^6^ CAR-T cells/kg dose. Encouragingly, in patients expressing the high level of BCMA on MM cells, 87% ORR and 73% CR were reported. Moreover, 100% ORR and 33% CR were detected in patients expressing a low level of BCMA on tumor cells [69].

#### CT103A anti-BCMA CAR-T cell

In another phase I trial study (ChiCTR1800018137), CT103A BCMA-targeted CAR-T cell, another next-generation CAR-T cell, was evaluated in nine patients diagnosed with MM. One to 6 × 10 6 cells/kg CAR-T cells were administered, resulting in 100% ORR and 67% CR [70].

### CD19-CAR-T cell

CD19 alludes to a signaling factor of a multimolecular complex on mature B cell and is also implied as a member of the immunoglobulin superfamily. It is considered an important and common target antigen-expressing on diverse B cell hematological malignancies, such as acute and chronic leukemias, and lymphomas [71]. As a result, since CD19 has an insignificant expression on MM cells, it is not a good choice for applying in MM patients. However, there is an apparent relationship between drug resistance related to BM microenvironment and CD19 expression in MM. Furthermore, CD19 expression has been evidenced on multiple myeloma stem cells (MMSCs), a population of MM tumors possessing the self-renewal and drug resistance capabilities [72, 73]. Thus, CD19 can be contemplated as a reliable target antigen for MM treatment strategy. It has been reported that following the administration of melphalan (high-dose) and autologous stem cell transplantation (ASCT), anti-CD19-CAR-T cell (CTL019) has emerged well-tolerated complete response in refractory MM cases [35]. Thus, CD19 can be considered a promise target antigen for MM treatment. Accordingly, a clinical trial study (NCT02135406) was conducted to evaluate the CTL019 therapy in 10 patients after ASCT and a high dose of melphalan. Concisely, the combinatorial treatment of ASCT and CTL019 led to a marked elevation of the progression-free survival (PFS) rates in MM patients with advanced stage [74] (Table 2).

**Table 2:** Non-BCMA-targeted CAR T cell clinical trials in multiple myeloma

Target antigen	Signaling domain	Clinical responses	Side effects	Conditioning	Registration code
CD19	4-1BB	ORR, 20% CR, 1 VGPR, 6 PR,2	Mild CRS Hypogammaglobulinemia Autologous GvHD	HDM + ASCT	NCT02135406
CD138	CD28	4 SD	Mild CRS	PCD, CP, or VAD	NCT01886976
	4-1BB	SD > 3 m, 4 Circulating PCL cells, 1	Infusion-related fever nausea and vomiting possible TLS	CP/Flu	?
	ND	PR,1	CRS grade 2	PCD, CP or VAD	?
Kappa LC	CD28	SD, 4	Mild CRS lymphopenia grade 3	CP or none	NCT00881920
NKG2DL	Dap10	ORR, 0%	Mild CRS	None	NCT02203825

*BCMA* B cell maturation antigen, *CAR* chimeric antigen receptor, *ORR* overall response rate, *CR* complete response, *PR* partial response, *VGPR* very good partial response, *SD* stable disease, *CRS* cytokine release syndrome, *HDM* high-dose melphalan, *ASCT* autologous stem cell transplantation, *PCD* pomalidomide-cyclophosphamide-dexamethasone, *CP* cyclophosphamide, VAD vincristine-doxorubicin-dexamethasone, *Flu* fludarabine, *GvHD* graft-vs.-host disease, *TLS* tumor lysis syndrome, *ND* no data, *NKG2D* natural killer group 2-member D

### CD19/BCMA CAR-T cell

In a clinical trial examination (NCT03196414) [75], Fu et al. administrated a third-generation CAR-T cell using anti-BCMA and anti-CD19 ScFv as an extracellular portion, and CD3ζ signaling domain accompanied by CD28 and OX40 costimulatory molecules in eight RRMM patients. Firstly, patients were treated with 1 × 107/kg CD19-CAR-T cells, and subsequently, they received 40% of BCMA CAR-T cells and 60% of the remaining cells on the next day. Similarly, in another study (SZ-MM-CART02 study, NCT 03455972) [76], Fu et al. administered the abovementioned CAR-T cells following the autologous transplantation as described above. The results showed approximately 100% ORR from the nine patients after treating with CAR-T cells.

Furthermore, in a phase II clinical trial study (ChiCTR-OIC-17011272) [77], twenty RRMM patients were treated by both humanized anti-CD19 CAR-T cells (1 × 106 cells/kg) and murine anti-BCMA CAR-T cells (1 × 106 cells/kg), resulting in 95% ORR.

### Kappa (κ) light chain-CAR-T cell

The light chain subsets (κ or λ) can be expressed on mature B cells and targeted by immunotherapy-based strategies. Therefore, in MM patients, a specified subset of the light chain can be targeted on MM tumor cells which would be different on healthy B cells. Immunoglobulins are not typically manifested on the plasma cell surface; howbeit, there is likely expression of immunoglobulins on MM stem cells [78]. In phase I of the clinical trial study, a light chain anti-kappa free monoclonal antibody, so-called MDX-1097, was assessed in patients with MM. Encouragingly, in one patient, a lower level of the serum-free light chain (FLC) was demonstrated, and also a complete metabolic reaction in another one was reported after the treatment. Also, 6 months period of stable disease (SD) was documented by phase II of the multiple-dose trial study in ten MM patients [79].

The light chain on MM cells can be remarkably targeted by CAR-T cells. Additionally, B cell depletion in different treatments of candidate patients will be led to the hard selection of a specific light chain for CAR-T cell therapy. In a phase I clinical trial study (NCT00881920) conducted by Ramos et al. [80], κ-CAR-T cell was engineered for recognizing the κ-light chain on tumor cells in seven MM and nine non-Hodgkin lymphoma patients. Before CAR-T cell therapy, patients treated with chemotherapy drugs were found to have no significant response against these drugs. After CAR-T cell therapy, 17 months of stable minimal residual disease, 2 years SD, and transient SD were reported among the MM patients; however, three patients indicated no objective responses to CAR-T cell therapy. Interestingly, severe CRS or other adverse effects were not demonstrated in patients following CAR-T cell therapy.

### NY-ESO-1-CAR-T cell

New York Esophageal Squamous Cell Carcinoma 1 (NY-ESO-1) refers to an intracellular oncoprotein that belongs to the cancer/testis (CT) antigens family. Also, NY-ESO-1 expresses in various cancers, like relapsed MM. In this regard, TCR-mimetic CAR-T cells have been engineered to target the Y-ESO1/HLA peptide complex [81, 82].

In a preclinical study, engineered NY-ESO-1-CART cells were administered to a mouse model with the NY-ESO-1/HLA-A2 MM, and evaluations revealed inhibitory effects on tumor cells. Interestingly, co-administrating the engineered T cells expressing NY-ESO1 and membrane-bound IL-15 could robust the anti-tumor cytotoxicity and persistence of memory CAR-T cells [81].

In a study conducted by Schuberth et al., anti-NY-ESO-1 CAR T cell was designed to distinguish the HLA-A*0201-NY-ESO-1157-165. Furthermore, MM cells that endogenously expressed NY-ESO-1 antigen were targeted by engineered CAR-T cells, redirecting to tumor targets. Through the anti-tumor cytotoxicity impact, CAR-T cells led to lysing the MM cells and secreting IFNγ. In addition to the effector role of T cells, NY-ESO-1 induced the generation of memory phenotype of T cells and IFNγ secretion [82]. In a phase I/II of the trial study, anti-NY-ESO-1 CAR-T cell efficacy was assessed after an autologous stem cell transplantation in twenty advanced MM cases. Findings revealed that about 80% of the patients showed appropriate clinical responses with 19.1 months median PFS [83].

### CD38-CAR-T cell

CD38 is a target antigen on MM cells known as a transmembrane glycoprotein which has several pivotal roles, including calcium regulation, signal transduction, and cell adhesion processes. Its expression is generally evidenced on NK cells, T cells, B cells, myeloid precursors, and plasma cells. Also, different healthy tissues have been shown to express comprising of osteoclast, guts, nervous system, prostate cells, and muscle cells [5, 84]. CD38 is intermediately expressed on both myeloma cells and healthy hematopoietic cells which elevates the risk of on-target/off-tumor toxicity [85]. Among the immunotherapy-based approaches, various mAbs have been developed to target CD38 on MM cells. Firstly, human anti-CD38 monoclonal named Daratumumab obtained FDA approval for treating RRMM alone or combinatorial with other medications. To eliminate the tumor cells, Daratumumab prompts the antibody-dependent phagocytosis, antibody-dependent T cell-mediated cytotoxicity (ADCC), and complement-dependent cytotoxicity (CDC) phenomena [86]. Moreover, isatuximab (SAR650984) is identified as another anti-CD38 mAb with potential anti-tumor cytotoxicity against MM [87].

According to the varying influences of mAbs on MM, there is a possibility to generate the anti-CD38 CAR-T cell. Previous studies have emerged that the anti-CD38-CAR T cells led to lyse the CD38^+^ MM cells with the capability of proliferation and cytokine production. Although using the anti-CD38-CAR-T cell has been shown to affect CD38^+^ MM cells, an undesirable on-target/off-tumor toxicity on CD38^+^ healthy hematopoietic cells and healthy tissues was reported. To prevent this toxicity, the light chain exchange strategy can be applied to generate the lower affinity ScFv. In this regard, it accurately targets only MM cells expressing a high level of CD38 without affecting normal cells with low CD38 expression [88]. Also, CD38 nanobody-based CAR-T cells are being generated and investigated in patients with MM. As a result, CAR-T cells demonstrated the effective anti-tumor function with slight on-target/off-tumor toxicity [89].

Moreover, caspase-9-based suicide genes can be used to solve this adverse effect of CAR-T cell [85]. As alternative approaches, using the all-trans retinoic acid or the histone deacetylase inhibitor panobinostat could selectively upregulate the expression of CD38 on MM tumor cells that might augment CAR-T cell cytotoxicity, and diminish the toxicity on healthy cells [90, 91].

To treat RRMM patients, anti-CD38-CAR-T cell monotherapy (NCT03464916) and combinational therapy targeting other molecules, including BCMA (NCT03767751), CD19 (NCT03125577), BCMA and NY-ESO-1 (NCT03638206), and BCMA plus CD138 or CD56 (NCT03473496, NCT03271632) have been investigated in several clinical trial studies.

### CD44v6-CAR-T cell

CD44 isoform variant 6 (CD44v6) glycoprotein is the main hyaluronan receptor that is commonly overexpressed on hematologic and epithelial tumors, evidenced in 43% of MM patients mostly in advanced and high-risk cases [92]. CD44v6 is a proper candidate for targeting by mAbs. In a phase I of the radioimmunotherapy trial study, bivatuzumab mertansine as a humanized anti-CD44v6 mAb indicated a protected consequence with merely reversible reactions in the skin [93]. In a study, Casucci et al. [94] have engineered the CD44v6-CAR-T cell to target tumor cells in patients with acute myeloid leukemia (ALL) and MM. The CD44v6-CAR-T cell that aimed to secrete IL-7 and IL-15 cytokines illustrated the greater anti-tumor function against MM cells with no toxic effects on normal keratinocytes and hematopoietic stem cells. Although reversible monocytopenia was reported as a main detrimental effect of CD44v6-CAR-T cell, it can be helpful to impede the CRS occurrence. For instance, suicide genes, including thymidine kinase gene and inducible caspase 9 gene, as safety switches can be administered to decrease CAR-T cell toxicity.

Moreover, a multicenter phase I/II of a clinical trial has been conducted by the EURE-CART project to investigate the CD44v6-CAR-T cell in acute myeloid leukemia (ALL) and MM patients [95] (see more details in https://www.eure-cart.eu/).

### CD56-CAR-T cell

CD56 belongs to the immunoglobulin superfamily and is identified as a cell surface glycoprotein with regulatory roles in cell-cell and cell-matrix interplay. CD56 is generally expressed on healthy activated T cells, NK cells, epithelial cells, and neural cells, but not on normal plasma cells. Furthermore, CD56 overexpression on tumoral plasma cells has been documented in over 78% of MM patients [2, 96]. Anti-CD56 monoclonal antibodies have been created to target CD56^+^ MM cells. For example, a humanized mAb called HuN901 demonstrated effective anti-tumor cytotoxicity in vitro and in vivo studies [97]. In a dose-escalation phase I trial study, lorvotuzumab mertansine (LM) that is considered an antibody-drug conjugate, has been evaluated against CD56^+^ tumor cells in 37 relapsed MM cases. Outcomes revealed the potent and well-tolerated anti-myeloma cytotoxicity impacts of LM solely, or along with lenalidomide and dexamethasone medications [98, 99]. However, there is the possibility of infection risks and infection-related deaths in using the LM, for it elicits the depletion of CD56^+^ immune effector cells [100]. In a preclinical study, Benjamin et al. [101] have designed the anti-CD56 CAR-T cell to target MM cells. Furthermore, CAR-T cell therapy has been investigated in clinical trial studies against CD56 in combination with other antigens that expressing on MM cells (NCT03473496 and NCT03271632). Since CD56 is expressed in central and peripheral nervous systems, neurologic toxicity may be considered a concern of using CD56-CAR-T cells.

### CD70-CAR-T cell

CD70 (CD27L) belongs to the tumor necrosis factor family that has a vital role in plasma cell differentiation. CD70 overexpression has been seen in solid tumors and hematological malignancies in comparison with a low expression on healthy cells [102]. CD70 is a potential target for mAb therapy. A humanized anti-CD70 mAb named CSGN-70 has been generated against MM cells based on anti-tumor cytotoxicity, and Fc-dependent antibody activity [102]. BMS-936561 and SGN-75 mAbs are the other examples of mAbs that have been produced to attack myeloma cells in MM cases [103, 104]. In a preclinical study, findings indicated that anti-CD70 CAR T cells could target the CD70^+^ MM cells with high efficacy and safety [105]. According to previously published data, the anti-CD70 CAR-T cell demonstrated potent functionality in both animal and human cancers in vitro [106, 107]. In a study performed by Shaffer et al. [108], an anti-CD70 CAR-T cell was engineered using the ScFv originated from CD27. Consequently, effective eradication of CD70^+^ MM cells and remarkable persistence of T cells were reported after CAR-T cell therapy. Despite the therapeutic application of anti-CD70 CAR T cells, it is not broadly utilized in MM patients. Thus, the expression of CD70 on MM cells appeared to be less and has variable grades [109].

### CD138-CAR-T cell

CD138 that called syndecan 1 is an adhesion molecule belonging to the syndecan family of heparan sulfate proteoglycans. It has a vital role in cell proliferation and the molecular adhesin process through binding to the collagen and fibronectin (extracellular matrix (ECM) molecules) [110, 111]. It can also bind to survival factors [112], such as cell proliferation-inducing growth factors and a proliferation-inducing ligand (APRIL) [112, 113]. Generally, mature epithelial and several malignant plasmas, as well as normal cells, could express CD138; however, it is not expressed on normal T and B cells [114]. Also, CD138 is overexpressed on MM cells, mostly in relapse or progressive disease that can lead to cancer progression [115]. Therefore, CD138 can be considered a potential and attractive candidate to target in MM patients. An anti-CD138 antibody-drug conjugate named BT062 (indatuximab) was applied as a therapeutic agent in MM patients. BT062 was tested in phase I/II of a clinical trial study, resulting in an objective clinical response in 1 patient out of 23 patients [116]. Similarly, the combination of BT062 with lenalidomide enhanced the overall response rate to approximately 83% [117].

Based on the accomplished preclinical examination, anti-CD-138 CAR-T cell therapy has been demonstrated a significant anti-tumor toxicity influence against myeloma cells in vitro and in vivo studies [118]. In a clinical trial study (NCT01886976), CD138-CAR-T cell therapeutic effect was evaluated in five RRMM patients following chemotherapy and stem cell transplantation. According to the findings, 3 to 7 months of SD, and a 10.5% to < 3% decrease in the number of MM cells were reported in four patients out of five patients who were administered with an average dose of 0.756 × 107 CAR-T cells/kg. Also, an increased frequency of CAR-T cells was identified after the first 2 months in the bone marrow [119].

Since the skin and mucosal toxicities were evidenced as adverse effects of CD138-CAR-T cells due to the broad expression of CD138 on normal epithelial cells, caution should be exercised in its application. Nonetheless, the safety of CD138-CAR-T cells and less toxicity on epithelial cells were found by a preclinical investigation [120].

Also, targeting CD138 by immune cells will confront problems due to the CD138 shedding from MM cells, and escaping from the immune system. This suggests using CD138-CAR-T cell in combination with CAR-T cell against other target antigens on the surface of the MM cells [121]. Consequently, different clinical studies have been planning to investigate the CD138-CAR-T cell therapy in combination with other CAR-T cells (NCT03196414, NCT03473496, and NCT03271632).

### SLAM7-CAR-T cell

SLAMF7 is a transmembrane receptor called CD319 or CS1 which belongs to the lymphocytic signaling activation molecule family [122, 123]. SLAMF7 contributes to phagocytosis of different hematopoietic malignant cells by macrophages [124]. Usually, SLAMF7 is expressed on a broad range of immune cells, including CD4 and CD8 T cells, NK cells, activated B cells, plasma cells, dendritic cells, and monocytes. SLAMF7 was first recognized as an NK cell receptor [125–127]. Also, SLAMF7 is a prevalent antigen is targeted by CAR-T cells. SLAMF7 has been shown to express remarkably on malignant plasma cells without expressing on hematopoietic stem cells and nonhematologic tissues. Generally, SLAM7 is also expressed in premalignant and new patients diagnosed with MM [126, 128]. Anti-SLAMF7 therapeutic antibodies have been developed, like humanized Elotuzumab mAb which obtained FDA approval to treat MM cases in combination with lenalidomide and dexamethasone medications [129]. In multiple clinical trial studies, anti-SLAMF7 CAR-T cells were investigated against myeloma cells. Anti-SLAMF7 CAR-T cells have engineered using the ScFv that derived from elotuzumab fused to the healthy donor and MM patients’ T cells. It has been reported that anti-SLAMF7 CAR-T cells could eliminate the primary and MM tumor cell lines, as well as normal lymphocytes with significant expression of SLAMF7 [125, 130]. In a clinical trial study (NCT03710421), SLAMF7-CAR-T cell containing the anti-SLAMF7 ScFv, memory-enriched T cells, and truncated EGFR (EGFRt) molecule was generated against MM cells. In this regard, It was observed that severe immune-mediated adverse events following administration of CAR T cells can be attenuated by cetuximab (EGFR monoclonal antibody) by inducing the suicide of CAR-T cell in an antibody-based-safety-switch manner [131].

UCARTCS1 is an example of an anti-SLAMF7 CAR-T cell that was designed for MM using the healthy-allogeneic T cell, and transcription activator-like effector nuclease (TALEN)-targeted gene editing. TALEN strategy shows a transient expression of endogenous, and restriction of SLAMF7-originated CAR-T cell fratricide. Based on the in vitro analysis, potent anti-tumor activity and MM cell lysis were reported using the UCARTCS [130, 132]. Other studies have determined the efficacy of either anti-SLAMF7 CAR-T cell or its dual form (SLAMF7/BCMA CAR) [124] along with its combination with other anti-MM drugs, such as daratumumab and lenalidomide [54, 133].

### Lewis Y-CAR-T cell

The carbohydrate antigen Lewis Y (LeY) alludes to a target antigen overexpressing around 50% of MM that correlated with the Lewis blood group antigen system. Interestingly, the lower expression level of the LeY on healthy cells makes it a suitable candidate for MM treatment [134]. According to previously published preclinical studies, LeY-CAR-T cells indicated a potential anti-tumor activity without on-target/off-tumor toxicity problem [135]. Moreover, anti-LeY CAR-T cell is underway the investigation in phase I of the clinical trial study (NCT01716364) which has not been reported in any results yet.

### NKG2D-CAR-T cell

Natural killer group 2-member D (NKG2D) is a transmembrane protein which has an activating role on cell surface receptor. Generally, NKG2D expresses on several known effector CD8 T cells, comprising of γδ T cells, NKT cells, and NK cells [136]. NKG2D detects multiple ligands, such as UL16-binding proteins (ULBP) and major histocompatibility complex class I polypeptide-related sequence (MIC) A/B. In response to infections, DNA damage, malignant transformations, and mentioned ligands will overexpress in tissues while the ligands are absent in physiological states. NKG2D expression has been reported in several solid and hematological cancers, such as AML and MM [137, 138]. Thus, NKG2D can be considered a potential target antigen for CAR-T cell therapy against MM. In a preclinical study conducted by Leivas et al. [139], NKG2D-CAR-NK cells demonstrated a potent eradication of tumor cells along with remarkable suppression of tumor growth; however, no effective responses were detected using NKG2D CAR-transduced T cells.

In the first human phase I clinical study, Baumeister et al. [140] have engineered the NKG2D-CAR-T cell to target several specific NKG2D ligands. The efficacy and safety of first-generation NKG2D-CAR-T cells were assessed in progressive RRMM and acute myeloid leukemia/myelodysplastic syndrome. Outcomes of the abovementioned study have implied that there was no prolonged persistence of CAR-T cell, objective clinical response, CRS, and neurotoxicity. Lymphodepletion is an essential step before CAR-T cell therapy is required to increase CAR-T cell engraftment [141, 142]. Accordingly, failure of this treatment in the mentioned trial study may be due to the use of first-generation CAR-T cell with limited cytotoxicity, and performing no lymphodepleting chemotherapy before CAR-T cell therapy.

### TACI-CAR-T cell

Transmembrane Activator and CAML Interactor (TACI) belong to the tumor necrosis factor receptor superfamily that is generally expressed at a lower level on malignant plasma cells [143]. TACI bounds to its ligand named APRIL which will be mediated by CD138 as a co-receptor [144]. APRIL is a common ligand for TACI and BCMA. In a preclinical study, Lee et al. [143] demonstrated that both BCMA^+^ TACI^+^ and BCMA^−^ TACI^+^ myeloma cells could be eliminated by APRIL-based CAR-T cells therapy. It means that APRIL-based CAR-T cells can lead to tumor death, albeit BCMA downregulation.

The clinical examinations have been conducted to evaluate the dual-APRIL-based CAR-T cells, developing to target both BCMA and TACI that expressed on myeloma cells (NCT03287804) [143, 145]. Moreover, according to recent research, T regulatory cells (Tregs) expressing TACI can be suppressed by APRIL-based CAR-T cells, leading to the eradication of MM cells in an indirect manner [146].

### GPRC5D-CAR-T cell

GPRC5D refers to a myeloma cell surface antigen that is a member of the human orphan family G protein-coupled receptor [147]. GPRC5D is generally expressed on CD138^+^ MM cells and hair follicle cells. Recently, GPRC5D has been considered an attractive target antigen for CAR-T Cell therapy in MM patients [148, 149]. Nevertheless, GPRC5D mRNA expression has only been detected on BM cells of MM cases, but its protein expression has not been found on myeloma cells by flow cytometry [150]. However, interestingly, GPRC5D expression on 98% of the CD138^+^ cells has been reported using the quantitative immunofluorescence method [149].

It has been proven that treating MM patients with BCMA-directed-CAR-T cell can be experienced with an antigen-loss relapse in the murine BCMA antigen escape model. This could improve using the GPRC5D-CAR-T cells [149, 151]. Accordingly, a phase I clinical trial study has been designed to investigate the efficacy of GPRC5D-CAR-T cell therapy in RRMM patients following the BCMA-directed-CAR-T cell therapy [149].

In another performed study by Smith et al. [149], engineered GPRC5D-CAR-T cell revealed powerful anti-MM responses against human MM cell lines (ffLuc^+^) xenografted in the NSG mice. Thereby, the GPRC5D-CAR-T cell would effectively contribute to MM treatment.

### Integrin-β7-CAR-T cell

Glycosylation or conformational modifications are considered post-translational processes which can convert the non-cancer-specific target epitopes to specific types. It significantly contributes to select appropriate target antigens in MM. Integrin-β7 is such an example of a post-translational MM target antigen that more than 10,000 hybridomas have been detected by screening against myeloma cells [152]. Among several designated therapies in MM patients, MMG49 mAb has been developed to potentially target a small fraction of CD19^+^ B cells and tumor-specific conformation of integrin-β7. Anti-integrin-β7 CAR-T cell constructed by MMG49 (derived from ScFv) was investigated against myeloma cells in vitro. This type of CAR-T cell could powerfully eradicate integrin-β7^+^ MM cells by secreting the IFNγ and IL-2 cytokines. Afterward, no tumor cell could escape, so rarely a malignant hematopoietic cell was seen [152].

### CD1d-CAR-T cell

CD1d is related to the MHC class I-like molecule known as another target antigen that is overexpressed on the premalignant and early stage of MM cells. In the advanced stage of the disease, although, the CD1d expression is decreased. CD1d presents the glycolipids to NKT cells, a similar way to the antigen-presenting cells mediate it by MHC [153]. In this regard, to target CD1d^+^ MM cells, CD19-CAR-NKT cell has been developed with the capability of targeting both CD1d and CD19 antigens on myeloma cells. This type of CAR-T cell indicated the efficient anti-tumor response in comparison with anti-CD19 CAR-T cell monotherapy. As an advantage, this anti-CD19 CAR-NKT cell therapy indicated no cytotoxicity on monocytes, resulting in an elevated expression of CD1d among blood cells [154]. It has been suggested that CD1d inducer drugs, such as ATRA and EZH2 inhibitors, would help to improve the efficacy of CAR-T cell therapy as a combinational treatment approach [154]. Consequently, engineering the NKT cell-based CAR-T cells can be a prominent approach to target CD1d^+^ MM cells in the early stages of the disease.

### CD299-CAR-T cell

CD299 receptor called SLAMF3, or Ly9, belongs to the SLAM family. Regardless of MM stage and contributed treatments on MM patients, CD299 is homogeneously and stably expressed on MM cells which is indispensable for MM cell survival [155–157]. It also has a positive expression on CD19-CD138-negative MM cells known as a drug-resistant MM cell population [157, 158]. From this point of view, CD299-targeted CAR-T cells can be generated to attack both masses of MM cells and remain MM cells which is resistant to chemotherapy. Firstly, in a preclinical study, Atanackovic et al. [158] have engineered CD229-CAR-T cell and investigated its functionality against MM cells. As a result, CD299-CAR-T cell has been applied potent anti-tumor cytotoxicity on malignant CD299^+^ MM cell lines with the least effect on healthy B and T cells.

## MM resistance or relapse after CAR-T therapy

Although CAR-T cell therapy may cause relapse or resistance events, this approach has a potentially curative impact to treat MM patients. Cancer cell development accompanied by a lower antigen expression and loss of antigen can be considered reasons for MM resistance or relapse following CAR-T cell therapy which has been documented in multiple preclinical and clinical studies [61, 159, 160]. As an example, the reduction of BCMA antigen on myeloma cells has been observed after CAR-T cell therapy. It can be suggested that targeting the multiple target antigens on tumor cells instead of only one antigen using the bi/tri-specific or multi-targeted CAR-T cells can solve the resistance/relapse problems. In a study by Yan et al., CD19/BCMA CAR-T cell as a dual-targeting CAR was evaluated in RRMM patients, which 100% overall response rate was reported after treatment [75].

The other cause of resistance/relapse in MM may be due to insufficient access of CAR-T cell to MM cells. MM cells have been emerged to locate in the microenvironment of bone marrow in the context of multiple extracellular matrix proteins which protect MM cells from CAR-T cell attack and diminish their accessibility to tumor cells [161]. Accordingly, in B cell lymphoma-bearing mice, CD19-CAR-T cell was traced whether reach to tumor site or not. Findings revealed that CAR-T cells were decoyed in the lungs instead of reaching the target site [162]. On the other hand, MM cells provide an appropriate condition to evade immune system responses, and CAR-T cell attack through the increased level of immunosuppressive factors and cells, including fibroblast growth factor (FGF), VEGF, stromal cell-derived factor (SDF)-1α, HGF, Treg cells, and myeloid-derived suppressor cells (MDSCs) [163–166].

As a postulation, CAR-T cell with no prolonged persistence may be a reliable reason for its insufficient efficacy that leads to MM relapse after CAR-T cell therapy. Based on some previous clinical trial studies in acute and chronic lymphocytic leukemia, there is a marked relationship between CD19-CAR T cell anti-tumor response, and CAR-T cell expansion and persistence [167, 168]. However, some others reported permanent anti-tumor responses, even the absence of CAR-T cells [169]. Also, the correlation between bb2121-CAR-T cell persistence and response has been evidenced in a previous study. As a result, in 20% of cases, over 1-year persistence was reported using bb2121-CAR-T cell. In this regard, a considerable frequency of CAR-T cells was found in patients treated with CAR-T cells after 28 days [63]. These described data are also consistent with MM patients achieving CAR-T cell treatment.

Moreover, MDSCs are identified as immunosuppressive cells leading to tumor progression. Augmented levels of MDSCs in the MM microenvironment decrease the immune factor infiltration into the tumor site and subsequently enhance the angiogenesis and tumor burden [170, 171]. In a combinational treatment of CAR-T cell along with NK cells has been shown that infusion of NK cells was led to MDSC eradication through targeting NKG2D and raised the survival of solid tumor bearing in mice [172].

## CAR-T cell side effects and safety increasing solutions

Like other cancer treatment approaches, adverse effects of CAR T cell therapy have been reported in several preclinical and clinical trial studies that cause the healthy tissue malfunction. Cytokine release syndrome (CRS), tumor lysis syndrome, neurotoxicity, anaphylaxis, prolonged cytopenia, hematological toxicity, on-target/off-tumor toxicity, disease resistance/relapse, insertional oncogenesis, and hypogammaglobulinemia are the common instances of side effects which mostly occur due to CAR-T cell over-activation.

In treated patients with MM by CAR-T cell, lower grades of CRS and neurotoxicity are considered the most prevalent adverse effects. Same as other malignancies, mentioned toxicities in MM patients could be controlled using the anti-IL-6 mAb (tocilizumab) and corticosteroids. Also, a T cell antigen coupler has been developed to recognize the target antigen in an MHC-independent manner and engage the naive TCR response, which has indicated lower toxicities than CAR-T cells [173].

The other well-known problem in MM CAR-T cell therapy is the absence of specific target antigens on tumoral plasma cells resulting in undesirable toxicities on healthy cells. Unlikely, the powerful response of CAR-T cells to healthy cells expressing a lower level of the target antigen elicits the on-target/off-tumor toxicity [174, 175]. To solve the problem, a proper target antigen or epitope should be identified by CAR-T cell.

Hence, some controllable regimens have been developed to mitigate the on-target/off-tumor toxicity, like the suicide switches mechanism. To overcome CAR-T cells over-activity problem, the inducible caspase 9 (iCas9) can be used as a proapoptotic suicide gene to protect healthy cells. iCas9 is dimerized and activates the apoptosis signaling through the administration of AP1903, a small switching molecule leading to CAR-T cell stops. iCas9 shows the 90% clearance of administered CAR-T cells following the AP1903 infusion after 12 h [176].

The RNA-based mechanism or bacterial-derived effectors can be applied as CAR-T cell manageable systems under the tetracycline-inducible promoter control. In the RNA-based system under the control of tetracycline, the secretion of IL-2 cytokine depends on the administration of doxycycline [177]. To trigger transient inactivation of the TCR signaling, OspF protein can be utilized as a bacterial-derived effector under control of tetracycline. This effect conducts the T cells to stop after doxycycline administration. These controlling regimens have not been carried out in using CAR-T cells [178]. Furthermore, exerting Ab-based switches, comprising of CD20, epidermal growth factor receptor (EGFR), and c-myc in CAR-T cell structure which can induce death in engineered T cells through the infusion of monoclonal antibodies against mentioned switches tags [179, 180].

Also, using the switch-on-based strategies to activate CAR-T cells in a controllable manner can promote the efficacy and safety of CARs. The split structure of CAR is constructed from separate activator and costimulatory portions, which can be assembled and activate the signaling through the administration of the small molecule. According to an investigation by Wu et al., a split anti-CD19 CAR-T cell was engineered, and outcomes indicated several efficient responses in vitro and in vivo examinations. This split strategy would be implemented in MM-targeted CAR-T cells to enhance the safety and efficacy [181].

Inhibitory CARs (iCARs) are another inhibitory mechanism that can halt CAR-T cell activity based on PD-1 or CTLA-4 inhibitory checkpoints. Recognizing the antigen that is expressed on healthy cells by iCAR induces the inhibitory signaling of CAR, which is specific to the target antigen on tumor cells [182]. The results of a meta-analysis demonstrated the potential effectiveness of CAR-T cell therapy in patients with hematologic malignancies; however, no overall signal regarding the relationship between CAR-T cells and solid tumor trials published so far. The results of this study can take part in making assistance to physicians, patients by determining the pros- and cons-associated with CAR-T cell therapy [183].

Finally, as a suggestion, using multi-targeted CAR-T cells and described inhibitory systems would be promising to robust CAR-T cell function. Exerting dual, tandem, or universal CARs has been shown to contribute to recognizing target antigens on tumor cells, simultaneously. They will be attractive due to provide an effective response without toxicity on healthy tissues [184–187]. However, the determination of proper and potent antigens on MM cells is a difficult challenge that affected the success of CAR-T cell therapy in MM patients.

## Allogenic CAR-T cells

Using allogeneic CAR-T cell therapy also may be useful in treating patients with progressive disease or for immediate necessary to CAR-T cells. In this case, engineering allogeneic CAR-T cell-derived from healthy donors would pave the patients’ necessity for available and feasible CAR T cell treatment. In the in vitro study and xenograft mice models, anti-BCMA allogeneic CAR T cell has been used to treat MM, which showed encouraging results. Since the high risk of graft-versus-host disease (GVHD) is a disadvantage of using this type of CAR-T cell, the TRAC gene has been removed from T cell by transcription activator-like effector nucleases (TALEN) editing strategy to minimize the GVHD risk [188]. Moreover, to perform selective lymphodepletion in a host, anti-CD52 mAb has been applied to knock out the CD52 gene in host lymphocytes [188]. Another example of applying the TALEN strategy to diminish the risk of GVHD is the SLAM7-CAR-T cell under clinical development that edited by eliminating the TRAC gene [132].

## The future direction of CAR-T therapy in multiple myeloma

Despite recent advances, there is no chemotherapy approach for patients suffering from multiple myeloma. In this regard, CAR-T cells are engineered T cells having lymphocyte-like signaling molecules which have been used not only in blood malignancies but also in various types of cancer against a range of target genes that are specifically expressed in malignant cells, so they have been introduced as a potential therapeutic goal to strengthen the immune system against malignant cells. Besides, CAR-modified T cell clinical trials have yielded unprecedented results in treating patients with MM and are expected to have a great influence by determining the suitable targets and reducing off-target effects [43]. The potency of CAR-T cell therapy in long-term multiple myeloma disease control, if fully comprehended, can have a transformative effect in this area. However, studies in the field of hematological malignancies are needed to identify new targets along with new combinational therapy. There are more than four hundred clinical trials focused on CAR-T cells in hematological malignancies along with the thousands of combinational therapy in this area. The challenge may be to determine better targets and/or most efficacious combinational therapy [43].

## Concluding remarks

In recent decades, cancer progresses occurred and substantial strides forward have been taken from investigations to encouraging clinical trials on the ground of cancer treatment. With the advent of ACT-based treatments, fighting against cancer has achieved impressive successes. CAR-T cell therapy has been considered a potent part of adoptive cell therapy-based approaches which has created a breakthrough for treating hematological and solid tumors using the modified recombinant target antigen receptor fused to T cells. Although there are various barriers responsible for CAR-T cell failure in solid tumors, promising results have been reported in hematological malignancies up to now.

There are multiple target antigens identified for targeting by CAR-T cells in hematological malignancies, in which anti-CD19-CAR-T cell has been known as the most used CAR-T cell. Multiple myeloma is a hematological malignancy defined by the increased level of transformed monoclonal plasma cells. Despite the considerable achievement of using therapeutic mAbs and drugs in MM, including bortezomib, carfilzomib, ixazomib, panobinostat, thalidomide, lenalidomide, pomalidomide, daratumumab, and elotuzumab, but most patients face relapse and resistance problems after these advanced treatments.

New horizons have been found for treating MM cases by the emergence of CAR-T cell therapy. MM has demonstrated promising reactions to CAR-T cell therapy, despite the difficulties in finding suitable target antigens. Based on several preclinical and clinical trial studies, multiple CAR-T cells have been developed to target different antigens expressing on myeloma cells.

As results of CAR-T cell therapy in vitro and in vivo investigations, potent anti-tumor cytotoxicity, cytokine production, and tumor escape prevention of CAR-T cells along with an encouraging overall response rate have been reported in most patients.

Among others, BCMA, CD19, CD38, and CD138 expressing on myeloma cells are mostly targeted by CAR-T cells. Anti-BCMA CAR-T cell has been mostly investigated in many investigations with encouraging implications against MM. Despite obtaining attractive and hopeful results, recognizing the specific target antigens, preventing the resistance/relapse after CAR-T cell therapy, and generating effective anti-tumor responses with high safety and efficacy remain as challenges in MM CAR-T cell therapy.

CRS and on-target/off-tumor toxicity have been indicated as the most common side effects of CAR-T cell therapy in MM. Accordingly, different approaches have been developed and applied to robust the safety and efficacy of CAR-T cells. Multi-targeted or bispecific CAR-T cells and inhibitory strategies, including suicide genes, Ab-based-switches, iCARs, and CRISPR/Cas9 systems are examples of safety solutions. Also, to provide a cheap, available, and a sufficient number of CAR-T cells for MM treatment, using allogenic CAR-T cells would be a helpful suggestion, particularly in progressive situations.

In conclusion, CAR-T cells by directing against a variety of targets can be utilized as warrior heroes for treating patients diagnosed with MM by the contribution of the other CAR-T cells or therapeutic approaches. However, conducting several investigations is critically required to recognize the predisposed and specific target antigens, modify the potent CAR-T cells, and improve the safety of CARs, which would be helpful to performing the impressive response of CAR-T cells against tumor cells.

## Data Availability

Not applicable.

## Abbreviations

CAR: Chimeric antigen receptor
GvHD: Graft-versus-host disease
CAR-NK: CAR-transduced NK cells
NK cells: Natural killer cells
ACTs: Adaptive cell therapies
NHL: Non-Hodgkin lymphomas
DLBCL: Diffused large B cell lymphoma
ALL: Lymphoblastic leukemia
MM: Multiple myeloma
AML: Acute myeloid leukemia
CRS: Cytokine release syndrome
CTLs: Cytotoxic T lymphocytes
iCAS9: Inducible caspase 9
