# Correction to: Any closer to successful therapy of multiple myeloma? CAR-T cell is a good reason for optimism

**DOI:** 10.1186/s13287-021-02523-2

**Published:** 2021-08-06

**Authors:** Faroogh Marofi, Safa Tahmasebi, Heshu Sulaiman Rahman, Denis Kaigorodov, Alexander Markov, Alexei Valerievich Yumashev, Navid Shomali, Max Stanley Chartrand, Yashwant Pathak, Rebar N. Mohammed, Mostafa Jarahian, Roza Motavalli, Farhad Motavalli Khiavi

**Affiliations:** 1 Department of Hematology, Faculty of Medicine, Tabriz University of Medical Sciences, Tabriz, Iran; 2 Department of Immunology, Faculty of Medicine, Zanjan University of Medical Sciences, Zanjan, Iran; 3 Department of Physiology, College of Medicine, University of Suleimanyah, Sulaymaniyah, Iraq; 4 Director of Research Institute “MitoKey”, Moscow State Medical University, Moscow, Russian Federation; 5 Tyumen State Medical University, Tyumen, Russian Federation; 6 Department of Prosthetic Dentistry, Sechenov First Moscow State Medical University, Trubetskaya St., 8-2, Moscow, Russian Federation 119991; 7 Department of Immunology, Faculty of Medicine, Tabriz University of Medical Sciences, Tabriz, Iran; 8 Immunology Research Center, Tabriz University of Medical Sciences, Tabriz, Iran; 9 DigiCare Behavioral Research, Casa Grande, AZ USA; 10 Faculty Affairs, Taneja College of Pharmacy, University of South Florida, Tampa, FL USA; 11 Faculty of Pharmacy, Airlangga University, Surabaya, Indonesia; 12 Bone Marrow Transplant Center, Hiwa Cancer Hospital, Suleimanyah, Iraq; 13 Toxicology and Chemotherapy Unit (G401), German Cancer Research Center, 69120 Heidelberg, Germany; 14 Stem Cell Research Center, Tabriz University of Medical Sciences, Tabriz, Iran; 15 Department of Virology, Pasteur Institute of Iran, Tehran, Iran

## Correction to: Stem Cell Research & Therapy (2021) 12:217 https://doi.org/10.1186/s13287-021-02283-z

The original article contained an error whereby Affiliation #5 was presented incorrectly. This has since been corrected.
