# Editorial Expression of Concern: Any closer to successful therapy of multiple myeloma? CAR-T cell is a good reason for optimism

**DOI:** 10.1186/s13287-025-04260-2

**Published:** 2025-04-10

**Authors:** Stem Cell Research & Therapy Editorial Board

**Affiliations:** The Campus, 4 Crinan Street, London, N1 9XW UK

**Editorial Expression of Concern: Stem Cell Research & Therapy (2021) 12:217** 10.1186/s13287-021-02283-z

The Editor-in-Chief of Stem Cell Research & Therapy is issuing an editorial expression of concern to alert readers that this article shows indication of irregularities in authorship during the publication process. The substantial authorship change that took place at revision does not appear to match the extent of the revision itself, and no sufficient rationale for the change has been provided by the authors. Navid Shomali, Roza Motavali did not state explicitly whether he agrees to this Expression of Concern. Faroogh Marofi, Safa Tahmasebi, Heshu Sulaiman Rahman, Max Stanley Chartrand, Rebar N. Mohammed, Yashwant Pathak and Roza Motavalli do not agree to this Expression of concern. Denis Kaigorodov, Alexander Markov, Alexei Valerievich Yumashev, Mostafa Jarahian and Farhad Motavalli Khiavi did not reply to correspondence from the Editor about this Expression of Concern.

